# Estimated Reduction in the Burden of Nontyphoidal *Salmonella* Illness in Canada Circa 2019

**DOI:** 10.1089/fpd.2022.0045

**Published:** 2022-11-10

**Authors:** Shiona Glass-Kaastra, Brendan Dougherty, Andrea Nesbitt, Mythri Viswanathan, Nadia Ciampa, Stephen Parker, Celine Nadon, Diane MacDonald, M. Kate Thomas

**Affiliations:** ^1^ Public Health Agency of Canada, Centre for Food-borne, Environmental & Zoonotic Infectious Diseases, Food-borne Disease and Antimicrobial Resistance Surveillance Division, Guelph, Canada.; ^2^ Public Health Agency of Canada, National Microbiology Laboratory, Division of Enteric Diseases, Winnipeg, Canada.; ^3^ Public Health Agency of Canada, Centre for Food-borne, Environmental & Zoonotic Infectious Diseases, Outbreak Management Division, Ottawa, Canada.

**Keywords:** *Salmonella*, burden of illness, interventions

## Abstract

Nontyphoidal *Salmonella* (NTS) is a leading cause of acute gastrointestinal illness in Canada, and reported cases have been on the rise since the early 2000s. To address this trend, agri-food industry partners and government have worked to identify and implement interventions, guided by the enhanced information provided by whole-genome sequencing, to reduce the incidence of NTS. A substantial reduction in the number of NTS cases reported occurred in 2019. Due to underreporting and underdiagnosis factors, the observed decrease in the number of reported cases represents a fraction of the true number of illnesses averted in the community. The objective of this study was to: (1) use burden of illness estimation methodologies to estimate the true number of NTS illnesses, hospitalizations, and deaths prevented, and (2) estimate the economic savings associated with the prevention of these cases. Compared with the previous 5 years, there were an estimated 25,821 fewer illnesses, 213 fewer hospitalizations, and 2 fewer deaths attributable to NTS in 2019. This corresponds to an estimated reduction of 26.9 million Canadian dollars in the economic burden of NTS. Although causality cannot be proven by this study, the findings are suggestive that the strategically implemented suite of public health actions, including genomic-based surveillance, policy changes, and interventions by the government and industry, were successful in reducing the economic and health burden of NTS infections in Canada.

## Introduction

Nontyphoidal *Salmonella* (NTS) causes a large burden of illness globally (Havelaar *et al.*, 2015). In Canada, NTS causes the second largest number of reported bacterial infections related to acute gastrointestinal illness, after *Campylobacter*. There were ∼7100 cases of NTS reported nationally each year from 2014 to 2017 (Public Health Agency of Canada, 2020). However, due to underreporting and underdiagnosis, this is an underestimate of the true burden of NTS illness in Canada. Previously, it was estimated that for every case of NTS reported in Canada, there are an estimated 26.1 cases in the population (Thomas *et al.*, 2013).

Canada observed an increasing trend in reported cases of NTS from the early 2000s through to late 2010s (Public Health Agency of Canada, unpublished data). *Salmonella* Enteritidis, the most commonly reported *Salmonella* serovar in Canada, was the predominant driver in this increase (Public Health Agency of Canada, 2019). The incidence of all other *Salmonella* serovars combined remained relatively constant through the same time period (Public Health Agency of Canada, 2019). National surveillance systems at the Public Health Agency of Canada (e.g., FoodNet Canada, the Canadian Integrated Program for Antimicrobial Resistance Surveillance, PulseNet Canada, National Enteric Surveillance Program) have been in place for nearly two decades to identify these trends, track potential sources, integrate work from multiple levels of government, and to link with the agri-food industry to inform interventions to improve the health of Canadians.

In recent years, there have been collective efforts by industry and government to address this increasing trend. These efforts were informed by advancements in laboratory methodologies, such as implementation of whole-genome sequencing (WGS), which have enhanced the ability of surveillance systems to make specific linkages between illnesses and sources over time (Nesbitt *et al.*, 2012).

A notable example of actions taken was the efforts taken to reduce the number of *Salmonella* Enteritidis infections attributable to frozen raw breaded chicken products, beginning in 2018. Numerous interventions were implemented including health risk assessments conducted by Health Canada; recalls by the Canadian Food Inspection Agency; public health notices and social media messaging by the Public Health Agency of Canada; public opinion research regarding frozen raw breaded chicken products; and targeted consumer awareness campaigns by Health Canada. Ultimately, the Canadian Food Inspection Agency implemented a directive to the industry to reduce *Salmonella* below detectable amounts in frozen raw breaded chicken products beginning April 1, 2019 (Government of Canada, 2018a). Some producers of frozen raw breaded chicken products were early adopters of processing interventions before that date.

**FIG. 1.**
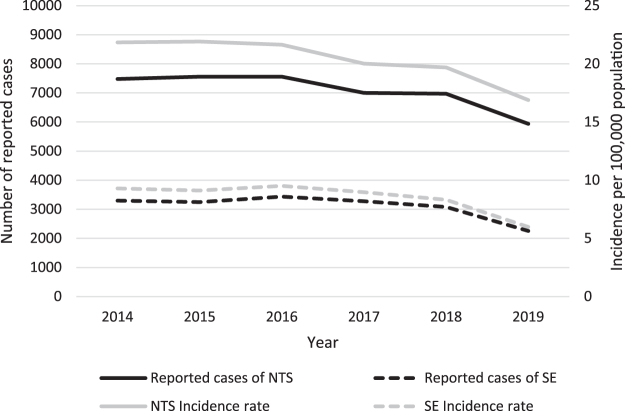
Case counts and incidence rates of NTS and *Salmonella* Enteritidis, as reported to the NESP 2014–2019. NESP, National Enteric Surveillance Program; NTS, nontyphoidal *Salmonella.*

In 2019, the reported cases to the National Enteric Surveillance Program indicate a 33% reduction in the incidence of *Salmonella* Enteritidis and a 16% reduction in the incidence of NTS, compared with 2017 (Fig. 1) (Public Health Agency of Canada, unpublished data). This is not a true measure of the reduced burden, however, due to underreporting and underdiagnosis factors inherent with enteric illness reporting (Thomas *et al.*, 2013). Burden of illness estimation methods allow us to account for such factors to estimate the total reduction in the number of cases, the economic burden associated with cases, and subsequently, any economic benefits.

This study estimates the reduction in the burden of *Salmonella* that occurred in Canada in 2019 in terms of the number of illnesses, hospitalizations, and deaths and by providing estimates of the economic savings associated with this reduction.

## Methods

We assessed the change in national reported rates of NTS in 2019 compared with the previous 5-year period (2014–2018). Underascertainment (i.e., underreporting and underdiagnosis) multipliers were applied and burden estimates were generated related to numbers of illnesses, hospitalizations, deaths, and associated costs to estimate the savings related to these public health interventions.

### Comparison of burden of illness and economic costs associated with NTS

The approach used by the U.S. Department of Agriculture Economic Research Service (Hoffmann *et al.*, 2012) provided the framework for the costing used in this estimate. To account for underascertainment, we used methodologies used in previous Canadian studies for estimating the number of NTS illnesses, hospitalizations, and deaths (Thomas *et al.*, 2013, 2015). In brief, the estimates were developed from stochastic models to consider the uncertainty of model inputs. Laboratory-confirmed cases of NTS, as well as hospitalizations and deaths attributed to NTS, were scaled up to account for underdiagnoses and underreporting. The economic burden estimate includes the costs of direct medical care, lost productivity, and premature mortality.

Three models were developed: background (2014–2018), NTS 2019, and reduction. The reduction model is the difference between the background and NTS 2019 models. The reduction model therefore describes the difference in the economic burden between these two time periods that is attributable to the reduction in NTS incidence in Canada. Details for the inputs and modeling approaches are provided in the appendices (Supplementary Appendix S1: Estimating Outcomes and Costs, Supplementary Appendix S2: Table of Model Inputs, and Supplementary Appendix S3: Québec Multiplier). The model outputs were described as mean values with 90% probability intervals (90% PI), generated using Monte Carlo simulation (100,000 iterations using @Risk software, an add-in for Microsoft Excel).

## Results

### Comparison of burden of illness and economic costs associated with NTS

The estimated number of illnesses related to NTS in 2019 was 70,833 (90% PI 49,594 to 98,667), with 1424 (90% PI 1069 to 1854) of those infected being hospitalized and 11 (90% PI 2 to 23) estimated deaths (Table 1). This was a reduction of an estimated 25,821 illnesses (90% PI −14,425 to 69,507), 213 (90% PI 26 to 420) hospitalizations, and 2 (90% PI 0 to 4) deaths compared with the annual averages from 2014 to 2018. These reductions translate to an estimated decrease in the economic burden associated with NTS of 26.9 (90% PI 1.8 to 56.6) million CAD (Table 2). Nearly half (48.9%) of the reduction in the economic burden is related to the estimated decrease in the premature mortality rate.

**Table 1.** Model Outputs Estimating the Annual Number of Illnesses, Physician and Emergency Room Visits, Hospitalizations, and Deaths Attributable to Nontyphoidal *Salmonella* in Canada

Model	Mean (90% PI)				
	Number of illnesses	Number of physician and emergency room visits	Number of hospitalizations	Number of workdays missed	Number of deaths
Background model (2014–2018)	96,654 (67,978 to 134,413)	29,657 (20,264 to 43,045)	1637 (1206 to 2162)	227,357 (155,415 to 322,779)	13 (2 to 27)
2019 model (2019)	70,833 (49,594 to 98,667)	21,562 (14,666 to 31,338)	1424 (1069 to 1854)	167,036 (113,963 to 237,321)	11 (2 to 23)
Reduction model^a^	25,821 (−14,425 to 69,507)	8189 (−6558 to 24,827)	213 (26 to 420)	60,411 (−33,890 to 164,672)	2 (0 to 4)

^a^ Because the reduction model is calculated using differences in distributions and generated using Monte Carlo simulation, the values will not always be the difference between the output values of the background and 2019 models.

PI, probability intervals.

**Table 2.** Model Outputs Estimating the Economic Burden Attributable to Nontyphoidal *Salmonella* in Canada (Millions of CAD [Adjusted to 2019])

Model	Mean cost in millions of CAD (90% PI)			
	Direct medical costs	Loss of productivity	Premature mortality rate	Total economic burden
Background model (2014–2018)	12.86 (9.74 to 16.65)	46.09 (31.50 to 65.43)	101.12 (15.46 to 209.92)	148.50 (61.44 to 258.28)
2019 model (2019)	11.36 (8.47 to 14.85)	33.86 (23.10 to 48.10)	88.34 (13.42 to 184.21)	123.08 (47.46 to 220.27)
Reduction model^a^	1.90 (0.39 to 3.57)	12.25 (6.87 to 33.38)	12.78 (0.47 to 33.13)	26.92 (1.65 to 56.56)

^a^ Because the reduction model is calculated using differences in distributions and generated using Monte Carlo simulation, the values will not always be the difference between the output values of the background and 2019 models.

PI, probability intervals.

## Conclusion/Discussion

This study explores the reduction in the burden of NTS that occurred in 2019 in terms of the number of illnesses, hospitalizations, and deaths, and provides estimates of the economic savings associated with this reduction. The mean outputs for the model found reductions in all measures, but the PI for the reductions in the number of illnesses and the number of physician and emergency room visits did include negative reductions (i.e., increases). Therefore, these findings should be interpreted with caution, as they signal that there is uncertainty in those model outputs.

It is estimated that there was approximately a 17.1% decrease in the economic burden of NTS in 2019 compared with the previous 5-year period, which corresponds to an overall estimated reduction of 26.9 million CAD in economic costs. Quantifying these reductions provides valuable insight into the benefits of reducing *Salmonella* infections.

In comparison with the reduction observed in Canada in 2019, FoodNet USA reported an incidence of 17.2 cases of *Salmonella* per 100,000 in 2019, which was higher than the 16.6 cases per 100,000 reported on average from 2014 to 2018 (Centers for Disease Control and Prevention, 2022). Furthermore, the European Food Safety Authority observed that the incidence of *Salmonella* in 2019, 19.5 cases per 100,000, was similar compared with the previous 3 years, which averaged an incidence of 19.7 cases per 100,000 (European Food Safety Authority and European Centre for Disease Prevention Control, 2021). The 19.6% reduction in Canada's NTS incidence rate in 2019 compared with the previous 5 years is therefore notable and is likely due to factors that are specific to Canada.

The collective actions taken by the industry and government, beginning in 2018, aimed at reducing the burden of *Salmonella* on Canadians are likely key contributing factors to this reduction. Before 2018, there were various interventions implemented to mitigate the harms of NTS, but the implementation of WGS of *Salmonella* for foodborne disease surveillance provided increased specificity when linking agri-food sources to human illness cases (Morton *et al.*, 2019; Government of Canada, 2020a, b). For example, with WGS implementation, human isolates collected by routine surveillance activities were able to be linked with specificity to frozen raw breaded chicken products of a particular production origin.

Previously, these cases would most likely only have been linked to ambiguous poultry exposure, rather than a definitive product. The ability to confidently attribute a significant number of NTS human cases to the consumption or handling of frozen raw breaded chicken products provided the rationale for government and industry to develop and implement targeted interventions.

Targeted interventions were also informed by public opinion research (Health Canada, 2018) and a national food consumption survey (Foodbook, April 2014–April 2015) (Public Health Agency of Canada, 2015), which included questions specifically aimed to better understand the knowledge and behaviors of Canadians with respect to frozen raw breaded chicken products. These studies confirmed that misperceptions were common in viewing these as precooked products requiring reheating only and that the degree of risk for a foodborne illness associated with them was low (Murray *et al.*, 2017). As a result, a consumer marketing campaign was launched by Health Canada in 2018–2019 that included digital advertising, awareness products, and messaging (Government of Canada, 2019a). Furthermore, increased risk communications through public health notices (Government of Canada, 2018c), food recall warnings (Government of Canada, 2019b), social media messages, and media engagement products were released (Government of Canada, 2018b, 2019c).

The continued link between these products and foodborne illness ultimately indicated that changes to the product itself were required to initiate substantial change in human illness. In July 2018, the Canadian Food Inspection Agency announced a new industry directive requiring the implementation of measures at the manufacturing/processing level by April 1, 2019, to reduce *Salmonella* to below detectable amounts in frozen raw breaded chicken products that are packaged for retail sale (Government of Canada, 2018a). This intervention led to decreased availability of uncooked product in the marketplace and, alongside surveillance data, indicates a reduction in the incidence rate of *Salmonella* Enteritidis and in the number of national *Salmonella* Enteritidis outbreak investigations in 2019. The authors hypothesize that these collective actions are likely at least partially responsible for the decline in the NTS incidence rate observed in Canada in 2019.

It had been long hypothesized that frozen raw breaded chicken products represented a unique and specific risk to consumers in Canada, with the lack of laboratory methods to differentiate strains contributing to the challenge (Hobbs *et al.*, 2017). Our findings are entirely consistent with the impacts of WGS implementation in other countries, for example, in the United States, the implementation of WGS alone prevents at least 25,000 foodborne illnesses per year with a health cost savings of 500 million U.S. dollars per year (Brown *et al.*, 2021).

An analysis of case studies on WGS implementation across public health laboratories in the United States, Canada, Italy, Argentina, and England found benefits including improved accuracy, better understanding of disease transmission, improved linkage of outbreaks that are related, improved information to inform control measures, and a reduction in disease burden (Alleweldt *et al.*, 2021). Thus, it is reasonable to conclude that the implementation of WGS in Canada was a seminal event that ultimately contributed to the reduction of illness and cost savings found in this study.

This study was not designed to measure the specific impact of all possible contributing factors to the reduction in NTS incidence rate. Statistical methods, such as time series analysis, could in theory have provided further evidence of a causal link between interventions and changes in the rate. However, due to there being multiple interventions that had varying and overlapping implementation dates, finding associations between any individual intervention and the reduction in NTS incidence was not possible. An additional challenge to evaluating the impact of the interventions was that the COVID-19 pandemic caused a substantial drop in the rate of NTS (and other enteric infections) in March 2020 and remained lower than historical averages for more than a year. This prevents any analysis from including data for 2020 or 2021, as it would require accurately adjusting for the impacts of COVID-19, which are not well understood at this point. It is possible that as rates stabilize at a “new normal” level, future analyses could explore the impacts of previous interventions.

A limitation of our model was that it was not capable of developing burden of illness estimates for specific serogroups of NTS. However, since the reduction in the NTS incidence rate was largely driven by a reduction in the incidence of *Salmonella* Enteritidis, general conclusions based on the wider NTS category are reasonable. As we gather additional data in the coming years on the impact of interventions, the next steps should include attempts to quantify the changes in the burden of illness by serogroup.

Ongoing surveillance, based on One-Health principles, is critical to increase the understanding of transmission pathways and sources of illness to identify interventions to reduce burden of illness. Overall, the study describes the estimated reduction in the burden of illness associated with *Salmonella* in Canada, and a concurrent reduction in costs due to the prevented illnesses, hospitalizations, and deaths. However, despite these reductions, salmonellosis remains the second leading bacterial pathogen causing reported gastrointestinal illness in Canada (Government of Canada, 2020b). Ongoing efforts are required to fill knowledge gaps, apply knowledge gained, and continue a collective approach to reducing the morbidity, mortality rate, and economic impacts of foodborne disease in Canada.

## Supplementary Material

Supplementary Appendix SA1

Supplementary Appendix SA2

Supplementary Appendix SA3
